# An Overview of the Molecular Mechanisms Contributing to Musculoskeletal Disorders in Chronic Liver Disease: Osteoporosis, Sarcopenia, and Osteoporotic Sarcopenia

**DOI:** 10.3390/ijms22052604

**Published:** 2021-03-05

**Authors:** Young Joo Yang, Dong Joon Kim

**Affiliations:** 1 Department of Internal Medicine, Hallym University College of Medicine, Gangwon-do, Chuncheon 24252, Korea; yjyang@hallym.ac.kr; 2 Institute for Liver and Digestive Diseases, Hallym University, Gangwon-do, Chuncheon 24253, Korea

**Keywords:** osteoporosis, sarcopenia, osteosarcopenia, chronic liver disease

## Abstract

The prevalence of osteoporosis and sarcopenia is significantly higher in patients with liver disease than in those without liver disease and osteoporosis and sarcopenia negatively influence morbidity and mortality in liver disease, yet these musculoskeletal disorders are frequently overlooked in clinical practice for patients with chronic liver disease. The objective of this review is to provide a comprehensive understanding of the molecular mechanisms of musculoskeletal disorders accompanying the pathogenesis of liver disease. The increased bone resorption through the receptor activator of nuclear factor kappa (RANK)-RANK ligand (RANKL)-osteoprotegerin (OPG) system and upregulation of inflammatory cytokines and decreased bone formation through increased bilirubin and sclerostin and lower insulin-like growth factor-1 are important mechanisms for osteoporosis in patients with liver disease. Sarcopenia is associated with insulin resistance and obesity in non-alcoholic fatty liver disease, whereas hyperammonemia, low amount of branched chain amino acids, and hypogonadism contributes to sarcopenia in liver cirrhosis. The bidirectional crosstalk between muscle and bone through myostatin, irisin, β-aminoisobutyric acid (BAIBA), osteocalcin, as well as the activation of the RANK and the Wnt/β-catenin pathways are associated with osteosarcopenia. The increased understandings for these musculoskeletal disorders would be contributes to the development of effective therapies targeting the pathophysiological mechanism involved.

## 1. Introduction

Osteoporosis is characterized by low bone mass, the deterioration of bone macro-and micro-architecture, and is a common complication observed in patients with chronic liver disease [1]. The prevalence of osteoporosis in chronic liver disease patients is 10–40%, which is higher than in the general population without liver disease [1]. The presence of osteoporosis in patients with liver disease adversely affects their clinical outcomes in terms of quality of life, survival, and liver-related complications, regardless of etiology and severity [1,2]. Since sarcopenia was first proposed as the concept of age-related loss of muscle mass in 1989, numerous studies have demonstrated its molecular pathogenesis and clinical implications, especially in the context of chronic liver disease, as the liver is an important organ for carbohydrate, protein, and lipid metabolism, whose deterioration results in protein supply dysregulation and hyperammonemia, inevitably influencing skeletal muscle homeostasis [3,4,5,6,7,8,9]. Therefore, sarcopenia was observed almost half of patients with liver cirrhosis, and negatively influenced the mortality and prognosis of liver disease [3,9]. Recent evidence of the interaction between osteoporosis and sarcopenia has led to the concept of osteosarcopenia, describing the concomitant development of sarcopenia and osteoporosis [10,11]. As common musculoskeletal complications that develop with aging, sarcopenia and osteoporosis share genetic, endocrine, and mechanical risk factors, and are also closely connected both mechanically and metabolically [12,13,14,15]. Osteosarcopenia has a negative effect on the quality of life and the clinical outcome in the events of falls, disability, hospitalization, and fracture, thus contributing to a higher mortality, which has highlighted its importance as a global health concern [16,17]. However, despite the high prevalence and clinical significance of osteoporosis and sarcopenia in patients with liver disease, attention and management strategies for these musculoskeletal disorders are frequently overlooked in clinical practice for patients with liver disease. Additionally, current understanding of molecular mechanism of osteosarcopenia in terms of bone and muscle crosstalk in patients with chronic liver disease is limited. Therefore, in this review, we describe what is currently known about the molecular mechanisms of osteoporosis, sarcopenia, and osteosarcopenia in chronic liver disease, to provide a comprehensive understanding of how these musculoskeletal disorders accompany the pathogenesis of liver disease and to generate interests in clinical implications of these musculoskeletal disorders in liver disease, which would be lead to promoting the clinical application of existing potential treatments, and further development of effective molecular targeted therapies for these musculoskeletal disorders in patients with liver disease.

## 2. Osteoporosis in Chronic Liver Disease

### 2.1. Prevalence and Clinical Outcomes of Osteoporosis in Chronic Liver Disease

A number of studies on osteoporosis in chronic liver disease have focused on cholestatic liver disease, including primary biliary cholangitis (PBC), primary sclerosing cholangitis (PSC), and end-stage liver cirrhosis [18]. The prevalence of osteoporosis is reported to be 20–32% in PBC and 15% in PSC, with the severity of liver disease being a risk factor for in PBC patients [19,20,21]. About 10–20% patients with PBC experienced facture, and the risk of fracture in this patient group was 2-fold higher than in the general population [22,23]. Patient with non-cirrhotic chronic hepatitis B or C exhibited lower bone mineral density (BMD) and developed osteoporosis with a prevalence of 10–30% [24,25,26,27,28,29]. Hansen et al. reported that fracture risk was higher in HCV-exposed patients [29]. In those with liver cirrhosis, the prevalence of osteoporosis was 12–28%, with advanced-stage cirrhosis and alcohol-associated liver cirrhosis being significantly associated with osteoporosis [30,31,32,33]. Interestingly, liver transplantation improved BMD, especially in patients receiving less glucocorticoid treatment, without cholestasis, and exhibiting an elevation of vitamin D and parathyroid hormones 4–6 months after liver transplantation [34,35]. With regard to non-cholestatic liver disease, a retrospective study reported that osteoporotic fractures were 2.5-fold more common in patients with non-alcoholic fatty liver disease (NAFLD), and low BMD was more prominent in those with higher disease activity, such as in cases with non-alcoholic steatohepatitis (NASH), significant fibrosis, and a high fatty liver index [36,37,38,39]. As alcohol consumption is an independent risk factor for osteoporosis, patients with considerable alcohol consumption developed osteoporosis without liver cirrhosis [40]. About 30% and 36% of alcoholic patients showed osteoporosis and vertebral fracture upon radiologic examination, respectively [41]. Importantly, abstinence increased BMD and bone formation marker osteocalcin levels in alcoholics [42]. Osteoporosis was observed in 25–34% of patients with hereditary hemochromatosis, independent of cirrhosis or hypogonadism [43,44,45]. The prevalence of osteopenia and osteoporosis was 9% and 50%, respectively, in patients with Wilson’s disease, which was significantly higher than in the healthy population [46]. In addition, Quemeneur et al. reported that half of patients with Wilson’s disease suffered peripheral fractures (Table 1) [47].

### 2.2. Molecular Mechanism of Osteoporosis in Chronic Liver Disease

The pathophysiology of chronic liver disease-associated osteoporosis is complex. Increased bone resorption is an important pathological characteristic of osteoporosis, especially in patients with end-stage cholestatic liver disease, viral hepatitis, and NAFLD. Suggested molecular mechanisms underlying bone resorption in chronic liver disease include the receptor activator of nuclear factor kappa (RANK)-RANK ligand (RANKL)-osteoprotegerin (OPG) system, upregulation of proinflammatory cytokines, such as interleukin-1 (IL-1), IL-6, and tumor necrosis factor alpha (TNF-α), as well as low levels of testosterone [1,2,48,49]. Bone remodeling is tightly regulated by osteocytes and osteoblasts through different cytokines and hormones modulating the activation, resorption, reversal, formation, and termination phase [50]. Various types of signals are involved, including mechanical strain, bone damage, as well as hormone-induced bone remodeling via macrophage colony-stimulating factor (M-CSF), RANKL, and OPG secreted by osteoblasts. RANKL and OPG are members of the TNF superfamily and are crucial for the regulation of bone resorption [51]. RANKL, through its receptor RANK, activates osteoclast formation, activation, and survival, while OPG, which is another receptor for RANKL, restrains osteoclastogenesis and inhibits bone loss by binding RANKL to prevent the RANK-RANKL cascade [51]. RANKL is a type II transmembrane protein with a C-terminal extracellular domain. This ectodomain cleaved is cleaved by matrix metalloproteinases to yield soluble RANKL (sRANKL) in the extracellular environment and both membrane-bound and sRANKL bind to RANK [52]. In chronic liver disease, an imbalance between RANKL and OPG leads to high sRANKL levels, which increases bone turnover. Thus, the ratio of OPG/sRANKL might indicate a homeostatic response for bone mass preservation [53]. Moschen et al. reported that sRANKL levels were higher in patients with liver disease than those in controls, except those in the cirrhotic subgroup, while OPG levels were found to be proportional to the severity of liver disease and highest in the cirrhotic subgroup with osteoporosis and osteopenia, resulting in a greater OPG/sRNAKL ratio in the cirrhotic subgroup with osteoporosis and osteopenia than in cirrhotic patients with normal BMD. These results suggested that high sRNAKL levels corresponded to increased bone turnover in patients with liver disease, and that OPG was also increased to compensate for negative bone turnover. Therefore, the high OPG/sRANKL ratio could be explained a response to maintain bone homeostasis in these patients [54]. In addition, RANKL/OPG gene expression, indicative of osteoblast-related osteoclastogenesis, was increased in the serum of jaundice patients [55].

In a chronic inflammation state, proinflammatory cytokines, especially IL-6, IL-1, and TNF-α, contribute to osteoclast activation and subsequent bone resorption [56]. IL-6 and IL-1 directly modulated osteoclastogenesis by enhancing osteoclast function [57]. They was also shown to indirectly promote osteoclast activity by facilitating RANKL production in osteoblasts [56,57,58,59]. Experimental studies revealed that IL-6 was associated with disrupted osteogenesis of bone marrow stem cells in osteoporosis models, and suppression of the IL-6 receptor prevented osteoclast-mediated bone resorption [60,61]. As IL-6 increases during liver injury to stimulate liver regeneration, upregulated IL-6 could affect bone remodeling in various types of liver disease [62,63]. Additionally, ethanol seems to activate osteoclasts through the induction of IL-6 and TNF-α [64,65]. Another potent inflammatory cytokine, TNF-α, is also involved in inflammatory bone resorption by stimulating RANKL expression in osteoblasts and tissue stromal cells, in turn promoting osteoclast differentiation and activity [66]. In particular, TNF-α enhanced CSF-1 receptor gene expression during the initial stage of osteoclastogenesis and subsequently stimulated osteoblast precursors, which resulted in increased osteoclast formation independent of the RANKL pathway [67]. These proinflammatory cytokines had effects on osteoporosis in viral hepatitis and NASH. Gonzalez-Calvin et al. showed that serum soluble TNF receptor p55 levels were significantly higher in patients with viral cirrhosis with osteoporosis than those without osteoporosis and positively associated with bone resorption [68]. In addition, since obesity is considered a chronic inflammatory state, these proinflammatory cytokines contribute to osteoporosis in NAFLD [69]. Indeed, low BMD is significantly associated with NASH presenting elevated alanine aminotransferase (ALT) and C-reactive protein (CRP) than in simple steatosis [37]. Another study showed that TNF-α levels are elevated in pediatric NASH [70]. Furthermore, Kim et al. reported that liver fibrosis in NAFLD is significantly correlated with low BMD, suggesting an association between aggravation of hepatic inflammation, fibrosis, and bone loss in NAFLD patients [38]. These proinflammatory cytokines have also been proposed as involved in dysbiosis-induced osteoporosis associated with chronic liver disease. Altered short-chain fatty acid (SCFA) levels and increased gut permeability (“leaky-gut syndrome”) may affect bone remodeling by regulating inflammation and immune system [2]. In addition, impaired liver function and cholestasis result in decreased 25-hydroxylation and intestinal absorption of vitamin D. Vitamin D deficiency is associated with osteoporosis in patients with liver cirrhosis [30,33]. Calcium and vitamin D deficiencies in patients with cholestatic liver disease caused secondary hyperparathyroidism, subsequently enhancing bone resorption [71]. Vitamin K is known to be involved in osteoblast apoptosis inhibition and osteoclast differentiation [72,73,74]. Therefore, decreased vitamin K levels were suggested to affect bone metabolism in chronic liver disease. Vitamin K deficiency reduced bone matrix proteins such as osteocalcin and osteonectin in patients with PBC [75,76].

Bone formation is also compromised in chronic liver disease as a result of toxic materials, sclerostin, and decreased anabolic hormones, which contribute to osteoporosis in patients with PBC, advanced stage liver cirrhosis, hereditary hemochromatosis, and Wilson disease [1,2,48]. Direct or indirect toxic compounds such as bilirubin, alcohol, iron, and copper accumulate in specific liver diseases, which can impair bone formation by inhibiting osteoblast proliferation and differentiation as well as bone mineralization by osteoblasts [45,55]. Unconjugated bilirubin decreased the survival of osteoblast, and osteoblast differentiation was significantly reduced only in jaundiced patients, except in patients with normal bilirubin levels, whereas ursodeoxycholic acid compensated for the negative effect of cholestatsis on osteoblast survival, proliferation and mineralization [55,77]. Previous studies reported that the severity of liver disease including cholestasis is significantly associated with osteoporosis in patients with PBC [19,20,23]. In hereditary hemochromatosis, lumbar spine BMD is significantly decreased in parallel to an increase of iron and alkaline phosphatase (ALP) levels [45]. In addition, bone synthesis is lower in alcoholic patients with low levels of osteocalcin, which is secreted from osteoblasts, and plays a role in calcium homeostasis, bone matrix mineralization, and osteoblastic proliferation [53]. Under physiological conditions, after bone resorption, mesenchymal stem cells and early osteoblast progenitors differentiate into osteoblasts through Wnt, bone morphogenetic protein (BMP), and fibroblast growth factor (FGF) signaling, leading to bone formation [2,78]. As a regulator of bone formation, sclerostin is produced in osteocytes and hinders osteoblast differentiation and proliferation, subsequently restricting bone formation by antagonizing Wnt signaling via binding to low-density lipoprotein receptor-related proteins (LRP) 5/6 transmembrane receptors [79,80]. As a result of sclerostin expression, the Wnt receptor is blocked and glycogen synthase kinase 3 phosphorylates β-catenin, which is involved in ubiquitination and degradation through the proteasome pathway [81]. Guanabens et al. proposed sclerostin as a crucial regulator of the Wnt/β-catenin pathway in relation to bone formation in patients with PBC [82]. A cross-sectional study revealed that sclerostin levels were significantly increased in patients with advanced cirrhosis when compared to those with early cirrhosis or healthy controls [83].

Insulin-like growth factor (IGF-1), which is secreted from hepatocytes by growth hormone (GH) has an anabolic effect on bone growth by suppressing osteoblast apoptosis and enhancing osteoblastogenesis through stabilization of the Wnt/β-catenin pathway [84,85]. In addition, IGF-1 reduced bone resorption through the OPG and RANKL system [86]. In end-stage liver disease, hepatocellular dysfunction and reduced GH receptors lead to low serum IGF-1 levels, subsequently causing osteoporosis [87]. Insulin, an important hormone associated with NAFLD, also affects bone remodeling by activating collagen synthesis and stimulating osteoblast proliferation and differentiation [88]. In addition, hypogonadism, which results from hyperestrogenism of portal hypertension in males and suppression of the hypothalamic-pituitary-gonadal axis in females, is frequently observed in hemochromatosis, liver cirrhosis, and alcoholics liver disease [1,89]. Since testosterone directly modulates osteoblasts and osteocytes via the androgen receptor to stimulate trabecular bone formation and prevent its loss, low testosterone level owing to hypogonadism in male enhances osteoclast function and induces bone turnover [53,90]. Though estrogen levels are increased in patient with liver cirrhosis due to increased peripheral conversion of androgen to estrogen, altered estrogen metabolism in liver cirrhosis contributes to a decrease in degradation of estrogen metabolites [91]. Because the bone protective effect of these estrogens is weak, it is not enough to overcome post-menopausal osteoporosis in women and liver-disease related osteoporosis in men [92,93].

## 3. Sarcopenia in Chronic Liver Disease

### 3.1. Definition of Sarcopenia

Sarcopenia, a condition characterized by the loss of skeletal muscle mass and strength, has been explored as a prognostic predictor for various diseases [94]. However, consensus criteria for the diagnosis of sarcopenia have not yet been established, and different definitions have been proposed by several groups [95,96]. The European Working Group on Sarcopenia in Older People (EWGSOP) first proposed the definition of sarcopenia in 2010, with muscle mass being a cardinal requirement for sarcopenia diagnosis [97]. The working group categorized sarcopenia into two categories. Primary sarcopenia referred to age-related muscle mass deterioration, while secondary sarcopenia was defined as caused by factors other than aging, such as inflammatory processes, disease-related disabilities, and inadequate energy or protein intake. Given the growing body of scientific data on sarcopenia, the EWGSOP recently updated diagnostic criteria so that muscle strength is the principal determinant for diagnosis with strict cut-off values (hand grip strength <27 kg for men and <16 kg for women) due to its significant correlation with clinical outcomes when compared to muscle mass [95]. According to the revised criteria, sarcopenia is defined as low muscle strength in parallel to low muscle mass or decreased muscle function. In contrast, the Asian Working Group for Sarcopenia (AWGS) regarded low muscle mass as the cardinal criterion for the diagnosis of sarcopenia. Therefore, AWGS defined sarcopenia as low muscle mass with low muscle strength or low physical performance [96]. Further, both EWGSOP2 and AWGS recognize entirely impaired muscle mass, muscle strength, and muscle function as diagnostic criteria for severe sarcopenia [95,96]. On the other hand, Clark et al. proposed in 2008 the concept of dynapenia, a state of age-associated decline in muscle strength which focused on other physiologic factors except for muscle mass loss in 2008 [98]. Therefore, dynapenia is similar to the revised EWGSOP definition of sarcoepnia in that decrease of skeletal muscle mass was not always necessary for the diagnosis of dynapenia, but sarcopenia is recognized to develop earlier in life, unlike dynapenia.

### 3.2. Prevalence and Clinical Outcomes of Sarcopenia in Chronic Liver Disease

Sarcopenia results from the accelerated loss of muscle mass and function, which in turn contributes to adverse clinical outcomes including fracture, frailty, and mortality. Although the prevalence of sarcopenia depends on the definition used, which is based on, for example, muscle mass cutoff points as well as other factors, the condition has been rigorously studied in the context of various chronic liver diseases (Table 2) [94]. With regard to non-alcoholic liver disease, previous studies reported a significant relationship between sarcopenia, sarcopenic obesity, and NAFLD, with this association being later described as independent of obesity and insulin resistance [99,100,101,102,103]. 

In addition, a longitudinal cohort study revealed that increased skeletal mass during the study period had a beneficial effect against the development of NAFLD or improved existing NAFLD at baseline [104]. Muscle mass loss was observed in 40–70% of liver cirrhosis patients, and most studies reported negative influences of sarcopenia on mortality and liver cirrhosis complications, such as hepatic encephalopathy [105,106,107,108,110,112,113]. The degree of sarcopenia was associated with Child-Pugh score in patients with cirrhosis, and sarcopenia was a predictor of survival independently or in combination with the model for end-stage liver disease (MELD) score, especially in patients with low scores (<15) [109,111,114]. Unfortunately, after liver transplantation, sarcopenia did not improve and even worsened because of immunosuppressive drugs such as steroids, calcineurin inhibitors, and mammalian target of rapamycin (mTOR) inhibitors [6]. Sarcopenia also had significant impacts on the development of diabetes mellitus, the risk of infection, the length of hospitalization, and mortality in patients who underwent liver transplantation [115,116,117,118,119,120,121,122]. Further, sarcopenia was shown to independently predict mortality, overall survival, and recurrence-free survival in patients with hepatocellular carcinoma [123,124,125].

### 3.3. Molecular Mechanism of Sarcopenia in Chronic Liver Disease

In normal physiology, skeletal muscle protein turnover is maintained as a balance between protein synthesis and breakdown. mTOR is an essential modulator of translational control, majorly involved in protein synthesis [126]. Exercise, branched chain amino acids (BCAAs), as well as various hormones, including testosterone, insulin, and IGF-1, activate the mTOR pathway in muscle cells through protein kinase B, which subsequently triggers several intracellular pathways for muscle protein synthesis, including p70 ribosomal S6 kinase 1 (S6K1) and eukaryotic initiation factor 4E binding protein (4E-BP1) [126]. In addition, the proliferation of muscle satellite cells, which are muscle fiber precursors, is critical for muscle growth and is activated in response to IGF-1 and BCAAs through protein kinase B [126,127].

In contrast, myostatin, a transforming growth factor beta (TGF-β) superfamily member produced in muscle, inhibits protein synthesis by maintaining satellite cells in a quiescent state, activating SMAD family transcription factors 2 and 3 (SMAD 2/3), and stimulating proteolysis via forkhead box O transcription factors (FoxOs) associated with the ubiquitin-proteasome pathway (UPP) and autophagy [128,129]. Impaired mitochondrial function, insulin resistance, and reactive oxygen species (ROS) also stimulate autophagy.

#### 3.3.1. Sarcopenia in Non-Cirrhotic Liver Disease

Sarcopenia and NAFLD share multiple pathophysiological mechanisms, including insulin resistance, chronic inflammation, cellular senescence, and oxidative stress [5]. As muscle cells are major targets of insulin as well as an essential amino acid reservoir for energy metabolism, sarcopenia affects various metabolic processes and is implicated in insulin resistance, NAFLD, and obesity [5]. The concept of sarcopenic obesity highlights this relationship [130]. With aging, muscle mass steadily decreases and is replaced by fat, which contributes to a reduction in physical activity and lower energy expenditure, especially the resting metabolic rate [131]. Therefore, muscle dysfunction and low muscle mass promote insulin resistance and NAFLD. In addition, physical inactivity combined with age-related comorbidity and estrogen changes during menopause contribute to NAFLD via fat mass deposition and sarcopenia [132].

In insulin resistance status, which is tightly associated with NAFLD pathogenesis, the inactivated insulin receptor pathway leads to a decrease in phosphorylated AKT (pAKT) levels, resulting in impaired protein synthesis, enhanced protein degradation and atrophy through autophagy and the UPP pathway [133]. As an anabolic hormone produced by hepatocytes and myocytes, IGF-1 stimulates muscle protein synthesis by activating the mTOR pathway through AKT phosphorylation and the suppression of FoxO1, muscle ring finger 1 (MuRF1), and the antrogin-1 pathway of protein catabolism [134,135]. Previous animal studies reported that serum and liver IGF-1 levels were decreased in fatty liver disease caused by insulin resistance, and NAFLD models with low IGF-I levels exhibited impaired muscle strength, function, and lower muscle fiber diameters [136].

NAFLD, insulin resistance, and obesity increase adipocyte lipolysis and have a stimulatory effect on immune cells such as macrophages and lymphocytes, leading to a chronic state of low-grade inflammation with increased production of proinflammatory cytokines, including TNF-α and IL-6. Shortly after exercise, IL-6 regenerates muscle cells through satellite cell regulation and glucose metabolism activation. In contrast, IL-6 promotes muscle catabolism in concert with TNF-α under chronic inflammatory states, such as infection and obesity [137,138]. Furthermore, TNF-α inhibits AMP-activated protein kinase (AMPK) signaling in muscle and insulin receptor autophosphorylation in fat tissue and the liver, which promotes insulin resistance [139,140]. Several adipokines, most notably adiponectin and leptin, are associated with muscle and liver metabolism. Adiponectin enhanced lipolysis, reducing inflammation and stellate cell activation in the liver, which in turn promoted glucose uptake and fatty acid oxidation in muscle [141,142]. Leptin activated stellate cells and fibrogenesis in the liver, while promoting IGF-1 activation and suppressing myostatin levels, which fostered myoblast proliferation and an increase in muscle mass [143]. In the state of insulin resistance, adiponectin is decreased, and leptin resistance is observed. Adipocytes were shown to activate the renin-angiotensin-aldosterone system (RAAS), in turn promoting insulin resistance and metabolic syndrome [144,145]. RAAS also influenced sarcopenia by inhibiting muscle regeneration and proliferation, while promoting muscle protein degradation via the UPP system [146,147]. Myostatin, a representative myokine related to sarcopenia, increased adipose tissue and suppressed adiponectin production as well as fatty acid oxidation in adipocytes. Myostatin knockout mice exhibited AMPK activation in the liver, adipocytes, and muscle, resulting in the restoration of insulin sensitivity [148,149,150,151]. Irisin, which is the cleaved extracellular domain of the muscle transmembrane fibronectin type III domain-containing protein 5 (FNDC5), increased during exercise, and promoted glucose uptake through the AMPK pathway [152,153]. Irisin also enhanced energy expenditure through uncoupling protein 1 (UCP1) activation and the browning of white fat in an animal model [153]. A recent study on single nucleotide polymorphisms reported the association between impaired irisin expression and severe hepatic steatosis [154]. Lee et al. reported an association between sarcopenia and NAFLD independent of insulin resistance or obesity, suggesting an effect of myokines on NAFLD [99].

#### 3.3.2. Sarcopenia in Liver Cirrhosis

The liver is an essential organ for carbohydrate, protein, and lipid metabolism. As the hepatic glucose reservoir is reduced, liver cirrhosis is regarded as a state of accelerated glucose starvation. In response, amino acids derived from skeletal muscle proteolysis are utilized for gluconeogenesis, which leads to the reduced BCAA levels observed in liver cirrhosis [155]. BCAAs including leucine, isoleucine, and valine, are essential for muscle mass preservation [155]. In particular, leucine activates protein synthesis via the mTOR pathway and is preferentially utilized for energy production in skeletal muscle [147]. Thus, the reduced BCAA levels in liver cirrhosis caused sarcopenia through muscle degradation [156]. Given that the liver is a crucial organ for ammonia disposal, the progression of portosystemic shunting and hepatocellular dysfunction in liver cirrhosis resulted in impaired ureagenesis and subsequent hyperammonemia [157]. In the hyperammonemic state, muscles convert ammonia to glutamate and glutamine via glutamine synthetase [158,159,160]. However, unless ureagenesis is recovered, no permanent ammonia disposal can occur, and circulating glutamine is just used in the process of ammoniagenesis, while hyperammonemia persists. Since carbon molecules from the tricarboxylic acid (TCA) cycle in muscle are relocated to other tissues through this pathway, bioenergetic dysfunction, impaired proteostasis, and sarcopenia develop in skeletal muscle [161]. Increased ammonia uptake in skeletal muscle contributes to molecular changes that favor sarcopenia, leading to a vicious cycle with decreased ammonia clearance and worsening liver cirrhosis outcome [161,162,163,164]. A large body of literature has proposed multiple pathophysiological mechanisms for hyperammonemia-induced sarcopenia. Qiu et al. demonstrated that serum and skeletal muscle ammonia levels were elevated in patients with cirrhosis, and hyperammonemia influenced myostatin expression via p65-NF-κB-dependent signaling [165]. Nishikawa et al. revealed that increased serum myostatin levels were associated with higher serum ammonia, a high Child-Pugh score, and were inversely related to psoas muscle mass [127]. In the hyperammonemia state, myostatin was suggested to inhibit protein synthesis not through the UPP system but via autophagy-mediated proteolysis [164]. Further, ammonia-associated mitochondrial dysfunction and increased ROS are regarded as potential triggers for the activation of autophagy [166]. Ammonia is converted to glutamate with the TCA cycle intermediate, α-ketoglutarate (α-KG) in mitochondria, followed by the SLC7A5-mediated conversion of glutamate to glutamine yielding leucine, which occurs in skeletal muscle [167,168,169]. Reduced α-KG from this reaction and hyperammonemia disrupted mitochondrial function and reduced adenosine triphosphate (ATP), with muscle mass and contractile function subsequently deteriorating [170]. The cellular stress response to reduced intracellular amino acid levels also influences sarcopenia. In alcoholic liver cirrhosis, phosphorylation and activation of general control non-depressible 2 (GCN2), an amino acid deficiency sensor, was reported [156]. Activation of GCN2 phosphorylated the alpha subunit of eukaryotic initiation factor 2 (eIF2a), which subsequently suppressed mRNA translation, impaired mTOR signaling, and suppressed protein synthesis [171,172]. Although the exact mechanism of GCN2 activation in hyperammonemia has not been elucidated, low BCAA levels have been proposed as potential mediators [173,174]. It is well known that testosterone increases muscle mass and enhances muscle protein synthesis through IGF-1 and subsequent mTOR activation [175,176]. Growth hormone and testosterone also contribute to sustained satellite cell activity by suppressing myostatin secretion and signaling [177,178]. However, increased serum aromatase activity in liver cirrhosis reduced serum testosterone levels, which was suggested as a potential contributor to sarcopenia, although whether these hormonal changes have an actual influence on protein synthesis and myostatin expression is unknown [179]. Recently, the gut microbiome has attracted major interest with regard to the pathophysiology of sarcopenia. In liver cirrhosis, compositional changes in the gut microbiota increased intestinal permeability and endotoxemia, while impairing SCFA production and antioxidant function [180,181,182]. These functional changes contributed to the development of sarcopenia through anabolic resistance, chronic inflammation with elevated IL-6 and TNF-α, altered mitochondrial function, as well as oxidative stress [3,183].

## 4. Osteosarcopenia

### 4.1. Prevalence of Osteosarcopenia

With a global increase in aging populations, evidence for osteosarcopenia in clinical practice is growing. In community-dwelling elderly adults, the prevalence of osteosarcopenia ranged from 18% in China to 40% in Australia [11]. In addition, patients with osteosarcopenia were more susceptible to fracture and frailty [16,184]. A study from Korea showed that 27.2% of elderly adults with hip fractures had osteosarcopenia, and these patients had a higher 1-year mortality rate (15.1%) compared to those with osteoporosis (5.1%) or sarcopenia (10.3%) alone [17]. Recently, several studies reported sarcopenia as a potential predictor of osteoporosis and reduced exercise tolerance in chronic liver disease, hepatitis C, as well as liver cirrhosis [185,186,187,188]. However, literature on the prevalence and clinical implications of osteosarcopenia in chronic liver disease remains limited. Seaki et al. reported that the prevalence of osteosarcopenia was approximately 20% in patients with chronic liver disease, with osteoporosis increasing the risk of vertebral fracture in these patients (Table 3) [189,190].

### 4.2. Crosstalk between Osteoporosis and Sarcopenia

**Figure 1:**
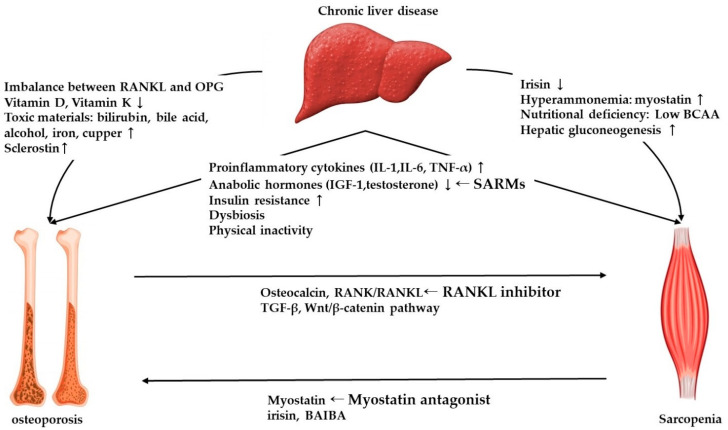
Overview of mechanism of osteosarcopenia in chronic liver disease and potential therapeutic strategies. RANKL, receptor-activator of nuclear factor kappa ligand; OPG, osteoprotegerin; IGF-1, Insulin-like growth factor-1; BCAA, branched chain amino acids; BAIBA, beta-aminoisobutyric acid; SARM, Selective androgen receptor modulator.

As discussed above, sarcopenia and osteoporosis share common risk factors and underlying pathophysiological mechanisms, including insulin resistance, decreased anabolic hormones, such as IGF-1 and testosterone, and upregulated inflammatory cytokines such as IL-1, IL-6, and TNF-α, which may contribute to the development of osteosarcopenia in liver disease, although their involvement has rarely been proven in osteosarcopenia models. Furthermore, sophisticated bidirectional crosstalk between muscle and bone consisting of not only mechanical interaction, but also paracrine and endocrine communication, plays a role in bone and muscle homeostasis (Figure 1). The mechanical perspective is based on the fact that muscle force is the major mechanical component to generate strain in bone. Therefore, decreased muscle function and performance result in decreased skeleton load and a subsequent deterioration of bone density. In addition, it is well known that muscle paralysis, atrophy, or immobilization promote both bone loss and osteoporosis [12]. However, sarcopenia does not always precede osteoporosis, and muscle mass changes alone cannot cause osteoporosis and vice versa.

Several mechanisms were proposed to underpin biochemical communication between sarcopenia and osteoporosis via the musculoskeletal secretome. Polymorphisms in the genes encoding glycine-N-acyltransferase (GLYAT), methyltransferase-like 21C (METTL21C), myostatin, α-actinin 3, proliferator-activated receptor gamma coactivator 1-alpha (PGC-1α), and myocyte enhancer factor 2C (MEF-2C) were related to bone and muscle loss [14,191]. In addition, peak muscle and bone volume in early life by genetic traits could postpone the time of sarcopenia and osteoporosis in geriatrics [10]. Guo et al. reported that *GLYAT* genes influence both bone and muscle remodeling through the regulation of glucose and energy metabolism [192]. Among METTL2 family of methyltransferase superfamily, METTL21C was proposed as having pleiotropic effects on bone and muscle through the NF-κB signaling pathway, which contributed to skeletal muscle atrophy [193]. Downregulation of this gene promoted reduced myogenic differentiation, impairment of calcium homeostasis in myocytes, and increased susceptibility of osteocytes to apoptotic agents such as dexamethasone [193]. MEF-2C, a transcription factor, which has been reported as a potential regulator of skeletal muscle gene expression, is also associated with bone homeostasis [194,195]. It regulates sclerostin, which inhibits osteoblastic bone formation, and modulates osteoclastic bone resorption. Further, deletion of the gene in osteocytes resulted in higher bone density [195].

As endocrine organs, muscles secrete various myokines, including myostatin, leukemia inhibitory factors (LIF), IGF-1, fibroblast growth factor 2 (FGF2), follistatin-like protein 1, brain-derived neurotrophic factor (BDNF), and irisin, which influence bone remodeling [196]. Myostatin acts as a negative regulator not only in muscle but also in bone by directly promoting the recruitment and differentiation of osteoclasts [197]. BMD was significantly increased in myostatin-deficient mice, and the use of myostatin inhibitors restricted bone loss as well as muscle loss in a number of musculoskeletal disease models [198,199,200]. Through its pro-myogenic effect, irisin also controlled bone mass by regulating energy metabolism and promoting osteoblast differentiation, resulting in increased bone cortical mass [201]. Recombinant irisin treatment increased cortical mineral density and bone strength in mice with an upregulation of pro-osteoblastic genes and a reduction of osteoblast inhibitors [202]. Recently, Kitase et al. demonstrated that beta-aminoisobutyric acid (BAIBA), a muscle metabolite secreted by contracting muscle, also played a role in preventing ROS-induced osteocyte cell death and preserved bone mass during immobilization [203].

Osteokines secreted from osteocytes, including osteocalcin, also have an influence on muscle. Osteocalcin produced in mature osteoblasts or osteocytes was involved in muscle mass remodeling by regulating glucose and energy metabolism as well as stimulating testosterone synthesis [204]. Mice deficient for GPCR6A, a G-protein-coupled receptor of osteocalcin, exhibited lower muscle mass. In contrast, mice lacking embryonic stem cell phosphatase, an enzyme that impairs osteocalcin activity, had increased muscle mass [204]. In addition, exogenously administered osteocalcin reversed the aging-associated decrease of exercise capacity and muscle strength in mice [205].

RANK is also expressed in skeletal muscle and suppresses skeletal muscle mass and function by restricting myogenic differentiation through the NF-κB pathway [206]. RANKL, an important modulator of bone resorption, was suggested to inhibit muscle function and mass, while RANKL inhibition ameliorated these effects, improving insulin sensitivity in osteoporotic mice and humans [207]. TGF-β is mainly produced from bone-forming osteoblasts and has an effect on perilacunar matrix modeling. In osteocytes, decreased TGF-β signaling caused bone fragility. TGF-β is also involved in bone-to-muscle communication [208]. Pathological TGF-β release from bone in breast cancer patients caused muscle weakness through reduced Ca^++^-induced muscle strength production [209].

Finally, the Wnt/β-catenin pathway regulates bone formation and is an important mediator of mechanical loading transmission at the bone surface [210,211]. Since secreted Wnt modulates cell proliferation, differentiation, and survival, the Wnt/β-catenin signaling pathway is known to play a significant role in skeletal muscle development, growth, and generation [212,213]. Huang et al. demonstrated that WNT3A produced by osteocytes promoted the differentiation of C2C12 myoblasts [214]. In addition, Wnt signaling is thought to be associated with satellite cell differentiation by promoting muscle regulatory factors during embryogenesis [12].

### 4.3. Management

#### 4.3.1. Exercise and Nutritional Support

Exercise may be considered for maintaining and improving skeletal muscle mass and BMD in chronic liver disease. Physical and electrical stimulation are known to positively influence osteogenesis during exercise [14]. A previous study reported the positive effect of active walking and strength training on BMD, and exercise could decrease the risk of falling and fracture in patients with osteoporosis [215,216]. In addition, several randomized controlled trials (RCTs) have shown that progressive resistance training stimulates muscle protein synthesis, enhancing muscle mass and strength in elderly patients with sarcopenia [217,218]. Roman et al. reported that combination treatment with moderate exercise and leucine supplementation increased exercise capacity and muscle mass in patients with liver cirrhosis [219]. Taken together, exercise-based treatment could improve skeletal muscle mass as well as bone density in patients with chronic liver disease. However, depending liver disease severity, some patients may experience obstacles in undertaking adequate exercise, particularly those suffering from end-stage liver disease.

For the nutritional support of the patient with osteoporosis, persistent calcium intake at a dosage of 1000 to 1500 mg/day as well as oral 25-hydroxy vitamin D supplementation of 400–800 IU/day or 260 µg every 2 weeks is recommended [2]. Since the disturbance of glucose and protein metabolism is a major pathophysiological mechanism of sarcopenia, dietary intervention and protein as well as BCAA supplementation are frequently proposed for the treatment of sarcopenia. Small frequent meals at short intervals instead of simply increasing caloric intake are conducive to increasing caloric intake and decreasing gluconeogenesis [6]. Tisen et al. showed that leucine-enriched BCAA supplementation significantly improved mTOR signaling and reduced muscle autophagy that contributes to enhancing muscle protein synthesis in patients with alcoholic cirrhosis. Also, L-leucine enriched BCAA supplementation reversed the GCN2-eIF2a phosphorylation and impaired mTOR signaling [171]. However, the beneficial effect of leucine-enriched BCAA treatment varied according to either accompanying improvements in metabolic parameters, or age [155,220]. Tryptophan, another essential amino acid, is associated with muscle mass homeostasis by regulating GH-IGF-1 signaling [221]. Though previous animal studies showed that a tryptophan-enriched diet promoted muscle protein synthesis in swine and tryptophan supplementation stimulated skeletal muscle signaling in vivo, which increased IGF-1, leptin as well as follistatin, and expression of myogenic genes in vitro, the effect of tryptophan treatment on sarcopenic patients with liver disease is rarely known [222,223]. In addition, glutamine, which plays an important role in protein metabolism, seems ineffective in preventing skeletal muscle loss in experimental studies [224,225,226]. Therefore, further investigations are warranted to determine if the clinical use of protein supplementation is effective for the treatment of sarcopenia in liver disease.

#### 4.3.2. Pharmacological Treatment

Although no pharmacological therapy has been approved for the treatment of patients with sarcopenia, effective osteoporosis therapeutics are well established. Widely used pharmacological treatments for osteoporosis include antiresorptive (denosumab, bisphosphate), anabolic (teriparatide, abaloparatide), sclerostin-inhibiting (romosozumab), and hormonal (hormone replacement therapy, selective estrogen receptor modulators) agents [78,227] (for a detailed review of these, see [78,227]) Regarding sarcopenia in chronic liver disease, few studies on ammonia lowering therapy reported positive effects on muscle mass increase. Kumar et al. reported that ammonia reduction using L-ornithine L-aspartate (LOLA) increased lean body mass, grip strength, and reversed myostatin levels as well as autophagy markers by reducing ammonia levels [228]. Rifaximin, which is a non-absorbable antibiotic used for the reduction of ammonia levels, was suggested to inhibit myostatin expression [229].

Recently, various novel treatments have been explored for therapeutically targeting not only bone, but also muscle. Denosumab, a humanized monoclonal antibody against RANKL, showed promising effects not only in protecting bone resorption and increasing bone mass but also by improving muscle strength and mass in postmenopausal women with osteoporosis [207]. A recent study of community-dwelling elderly adults reported that denosumab significantly improved balance, fear of falling, and physical function compared to zoledronic acid treatment [230]. Denosumab may thus have potential as a osteosarcopenia therapeutic, and further studies are necessary to confirm its effects on muscle mass and function.

Although testosterone has an anabolic effect on both bone and muscle, clinical studies have rarely reported the benefit of testosterone treatment on musculoskeletal disease. Selective androgen receptor modulators (SARMs) exhibited anabolic effects on muscle and bone. In addition, since SARMs selectively target tissue, there were little androgenic side effects [231]. In a recent phase II trial, VK5211, Ristic et al. reported that oral non-steroid SARMs improved lean muscle mass and increased bone formation with procollagen type 1 N propeptide (P1NP), which highlights its potential for the treatment of osteosarcopenia [11].

Targeting myostatin, which is an important negative regulator of bone and muscle, has also been rigorously studied. ACE-031 is a soluble form of activin receptor type IIB, which binds myostatin to neutralize its effects. A previous study showed that ACE-031 treatment was well tolerated and increased bone formation markers, improving lean body mass in postmenopausal women [232]. In a phase II trial in elderly adults with a history of falling, treatment with a humanized myostatin antibody increased lean body mass and improved performance-based measures associated with muscle strength [233]. However, as myostatin is also present in cardiac tissue, myostatin targeted treatment may cause adverse events such as cardiomyopathy [234]. Therefore, further studies to clarify the efficacy and long-term safety of myostatin inhibition beyond experimental studies are warranted before its clinical use. Other pharmacological treatments, including growth hormone and IGF-1, have been investigated for the treatment osteoporosis and sarcopenia, but beneficial effects were not observed [14]. We summarize current pharmacologic treatments of osteoporosis, sarcopenia, and osterosarcopenia in Table 4.

## 5. Conclusions

A number of previous studies have demonstrated that osteoporosis and sarcopenia are common musculoskeletal disorders which negatively affect the quality of life, and increasing morbidity and mortality in patients with chronic liver disease. However, the awareness of these musculoskeletal disorders in liver disease is frequently neglected in clinical practice and current management guidelines for various liver diseases such as viral hepatitis, decompensated liver cirrhosis did not state the managements for these musculoskeletal disorders. In this review, we addressed the molecular mechanisms of osteoporosis, sarcopenia, and osteosarcopenia in chronic liver disease, to increase the understanding and attentions of these musculoskeletal disorders, especially in liver disease. Increased bone resorption through the receptor activator of nuclear factor kappa (RANK)-RANK ligand (RANKL)-osteoprotegerin (OPG) system and upregulation of inflammatory cytokines are considered important mechanisms for osteoporosis in non-alcoholic fatty liver disease (NAFLD) and viral hepatitis. Osteoporosis in cholestatic liver disease is associated with impaired bone formation, increased bilirubin and sclerostin, and lower insulin-like growth factor-1. Sarcopenia in NAFLD is associated with insulin resistance and obesity, whereas in liver cirrhosis, it is influenced by hyperammonemia, low amount of branched chain amino acids, and hypogonadism. In addition, osteoporosis and sarcopenia shared common underlying mechanisms, including insulin resistance, decreased anabolic hormones, such as IGF-1 and testosterone, increased inflammatory cytokines, including IL-1, IL-6, and TNF-α, as well as dysbiosis. Moreover, biochemical crosstalk between bone and muscle through various signaling pathways have been elucidated, highlighting osteosarcopenia as a prevalent combination of osteoporosis and sarcopenia. Given the significant roles of these musculoskeletal disorders as prognostic predictors and their major involvement in liver disease pathogenesis, increased knowledge for the molecular mechanism of these musculoskeletal disorders could be contribute to improvement of not only musculoskeletal disorder itself, but also prognosis of liver disease by promoting the clinical application of existing potential therapeutics for these musculoskeletal disorders in patients with liver disease, and further development the effective therapies targeting the pathophysiological mechanism involved. 

## Figures and Tables

**Table 1:** Representative clinical studies for osteoporosis in chronic liver disease.

Studies on Patients with Primary Biliary Cholangitis (PBC) or Primary Sclerosing Cholangitis (PSC)					
Author, Year	Study Aim	Study Design	Study Population	Method to Diagnosis Osteoporosis	Outcome
Menon et al., 2001 [19]	To evaluate the prevalence and risk factor of bone disease in patients with PBC and to determine the rate of bone loss over time.	Retrospective study	176 patients with PBC	DEXA	The prevalence of osteoporosis is 20% in patients with PBC. Age (OR 1.2 (1.1–1.2)), BMI (OR 0.8 (0.7–0.9)), advanced stage (3 or 4) (OR 6.3 (1.8–21.6)), and history of fractures (OR 4.1 (1.0–16.8)) were independent indicators of osteoporosis. Serum bilirubin level was independently associated with the rate of bone loss over time.
Guanabens et al., 2005 [20]	To find out the prevalence and risk factors for osteoporosis in women with PBC	Cross-sectional study	142 women with PBC and 1305 age-matched control subjects	DEXA	Prevalence of osteoporosis was higher in PBC (32.4%) than in normal women (11.1%). Older age, higher Mayo risk score, lower BMI and advanced histological stage were independent risk factors for osteoporosis.
Solaymani-Dodaran et al., 2006 [22]	To quantify the excess fracture risk in people with PBC	Retrospective cohort study	930 patients with PBC and 9202 age- and sex-matched control subjects.	NA	There were approximately 2-fold relative increases in the risk of any fracture (HR 2.03 (1.70–2.44)), hip fracture (HR 2.14 (1.40–3.28)), and ulna/radius fracture (HR 1.96 (1.42–2.71)) for the PBC cohort compared with the general population.
Guanabens et al., 2010 [23]	To assess the prevalence and risk factors for fractures and the fracture threshold in women with PBC	Prospective study	185 women with PBC	DEXA	The prevalences of vertebral, non-vertebral, and overall fractures were 11.2%, 12.2%, and 20.8%, respectively. Vertebral fractures, are associated with osteoporosis (OR 8.48 (2.67–26.95)). Osteoporosis and osteopenia are associated with the severity of liver damage.
Angulo et al., 2011 [21]	To identify prevalence and rate of progression of bone disease in patients with PSC and to identify predictors of bone disease and progression.	Retrospective longitudinal cohort study	237 patients with PSC	DEXA	Osteoporosis was found in 15% of patients (RR 23.8 (4.6–122.8)). Old age (OR 7.8 (3.3–18.3), BMI (OR 4.9 (1.9–12.6), and long duration of inflammatory bowel disease (OR 3.6 (1.5–8.4)) correlated with the presence of osteoporosis.
**Studies on Patients with Viral Hepatitis**					
**Author, Year**	**Study Aim**	**Study Design**	**Study Population**	**Method to Diagnosis Osteoporosis**	**Outcome**
Schiefke et al., 2005 [28]	To evaluate BMD and bone turnover markers in patients with non-cirrhotic CHB or CHC	Cross-sectional study	43 patients with HCV (*n* = 30) or HBV (*n* = 13) infection without histological evidence for liver cirrhosis.	DEXA	Osteoporosis is observed in 32% of non-cirrhotic CHB or CHC patients. Altered bone metabolism with increased bone-specific ALP and iPTH already occurred in advanced liver fibrosis without cirrhosis.
Orsini et al., 2013 [24]	To identify the prevalence of osteoporotic vertebral fractures and low BMD measurements in men with non-cirrhotic CHC.	Cross-sectional study	60 non-cirrhotic CHC patients and 59 healthy controls	DEXA	Non-cirrhotic untreated CHC patients have lower BMD at the femur as compared to healthy men in spite of the absence of significant bone and mineral abnormalities.
Hansen et al., 2014 [29]	Comparison of fracture risk between HCV-seropositive (HCV-exposed) patients and the general population, and between patients with cleared and CHC infection.	Retrospective cohort study	12,013 HCV-exposed patients from the Danish HCV cohort, and 60,065 general population	NA	HCV-exposed patients had increased risk of all fracture types (adjusted incidence rate ratio (aIRR) 2.13–2.18) whereas overall risk of fracture did not differ between patients with chronic vs. cleared HCV-infection.
Lai et al., 2015 [25]	Association between BMD, systemic inflammation, and markers of bone turnover in CHC without cirrhosis	Cross-sectional study	60 non-cirrhotic CHC patients	DEXA	Low BMD was observed in 42% (30% had osteopenia, 12% had osteoporosis) of non-cirrhotic CHC patients, but not associated with systemic inflammatory markers. Patients with low BMD had higher serum phosphorus and pro-peptide of type 1 collagen.
Huang et al., 2017 [26]	To assess BMD and prevalence of osteoporosis in CHB patients	Case-control study	148 CHB patients and 148 age- and gender-matched healthy controls	DEXA	The prevalence of osteoporosis in either of lumbar spine, total hip or the femur neck was significantly higher in the CHB patients group (12.8%, 11.5%, 12.2%) compared with the healthy control (4.7%, 4.1%, 4.7%). CHB infection was associated with low BMD and increased the risk of osteoporosis.
Wei et al., 2019 [27]	To identify the effect of ETV and TDF on the development of osteopenia/osteoporosis	Retrospective cohort study	1224 Asian CHB patients	DEXA	There is no significant increase in the incidence of osteopenia/osteoporosis for patients with CHB treated with TDF (HR 0.74 (0.34–1.59)) or ETV (HR 0.98 (0.51–1.90)) during a median follow-up of about 4 to 5 years.
**Studies on Patients with Liver Cirrhosis (LC)**					
**Author, Year**	**Study Aim**	**Study Design**	**Study Population**	**Method to Diagnosis Osteoporosis**	**Outcome**
Monegal et al. 1997 [33]	To find out the prevalence and risk factor of bone disease in patients with end-stage liver disease waiting for OLT.	Prospective study	58 cirrhotic patients	DEXA	43% patients had osteoporosis and vitamin D deficiency, reduced PTH levels, and hypogonadism are observed in cirrhotic patients. Alcoholic and Child-Pugh C patients showed the lowest femoral BMD.
Sokhi et al., 2004 [31]	To assess the BMD in different subgroups among pretransplant cirrhotic patients.	Retrospective study	104 cirrhotic patients	DPA	The overall prevalence of osteopenia and osteoporosis were 34.6% and 11.5%, respectively, being significantly higher in females than in males. BMD is significantly lower in those with CTP class C than those with CTB class B in both males and females.
Goubraim et al., 2013 [30]	To evaluate prevalence and risk factors for metabolic bone disease in patients with viral cirrhosis	Prospective study	46 cirrhotic patients	DEXA.	Osteopenia and osteoporosis is observed in 52.2% and 28.2% patients, respectively. There was no independent factor associated with bone disorders although bone disorders were significantly more frequent in old patients with low BMI, long duration of liver disease, and low vitamin D level.
Zheng et al., 2018 [32]	To evaluate osteoporosis or osteopenia in patients with cirrhosis	Retrospective study	217 LC patients and 229 subjects without liver diseases	DEXA	Osteoporosis was found in 20.3% and older age (OR 1.78), lower BMI (OR 0.63), greater fibroscan score (OR 1.15), and alcoholic liver cirrhosis (OR 3.42) were independently associated with osteoporosis in cirrhotic patients.
**Studies on Patients Who Underwent Liver Transplantation**					
**Author, Year**	**Study Aim**	**Study Design**	**Study Population**	**Method to Diagnosis Osteoporosis**	**Outcome**
Monegal et al., 2001 [34]	To determine the incidence and risk factors of skeletal fractures and to analyze the long-term evolution of bone mass, bone turnover and hormonal status after LT	Prospective study	45 patients following LT	DEXA	Fifteen patients (33%) developed fractures after liver transplantation, and pre- transplant risk factors for fractures were age and low bone mass (OR 5.69 (1.32–24.53)). Bone mass decreased during the first 6 months and after then bone formation parameters is increased.
Guichelaar et al., 2006 [35]	To identify the prevalence and predictive factors for low bone mass before OLT, Posttransplant bone loss, and bone gain at the lumbar spine with long-term follow-up after OLT	Prospective cohort study	360 patients with end-stage PBC and PSC	DPA & DEXA	Most patients (77%) with advanced PBC and PSC have osteopenic bone disease, and risk factors for hepatic osteopenia are low BMI, older age, postmenopausal status, the presence of muscle wasting, high ALP, and low serum albumin. After OLT, aggressive bone loss occurs during the first 4 months, with risk factor of younger age, PSC, higher pretransplant BMD, no IBD, shorter duration of disease, current smoking and ongoing cholestasis at 4 months. After the first 4 postoperative months, bone gain occurs during the first 2 years with favoring factors for improvement of lower baseline and/or 4-month BMD, premenopausal status for females, lesser glucocorticoids, no ongoing cholestasis, and higher levels of vitamin D and parathyroid function.
**Studies on Patients with Non-Alcoholic Fatty Liver Disease (NAFLD)**					
**Author, Year**	**Study Aim**	**Study Design**	**Study Population**	**Method to Diagnosis Osteoporosis**	**Outcome**
Li et al., 2012 [36]	Association between NAFLD and osteoporotic fracture	Cross-sectional study	7797 Chinese adults (including 2352 patients with NAFLD)	NA	The prevalence of osteoporotic fractures was significantly higher in men with NAFLD (3.6 vs. 1.7%), and the presence of NAFLD was significantly associated with osteoporotic fracture among men. (OR 2.53 (1.26–5.07))
Purnak et al., 2012 [37]	Association between BMD and liver function in patients with NASH.	Cross-sectional study	102 patients with NAFLD and 54 healthy controls	DEXA	The presence of elevated serum ALT and hs-CRP levels, which are suggestive of NASH, was associated with lower BMD although simple steatosis of the liver does not affect BMD.
Kim et al., 2017 [38]	Association between liver fibrosis and BMD in patients with NAFLD	Retrospective cross-sectional study	231 subjects (including 129 patients with NAFLD)	DEXA	Significant liver fibrosis was independently associated with overall osteopenia and osteoporosis in subjects with NAFLD. (OR 4.10 (1.02–16.45)).
Ahn et al., 2018 [39]	Association between fatty liver index (scoring model for NAFLD) and BMD	Population-based, cross-sectional study	4264 adults	DEXA	Fatty liver index was negatively correlated with total hip (*p* = 0.004), femoral neck (*p* < 0.001), and whole body BMD (*p* = 0.01) in men independent of insulin resistance.
**Studies on Alcoholics**					
**Author, Year**	**Study Aim**	**Study Design**	**Study Population**	**Method to Diagnosis Osteoporosis**	**Outcome**
Peris et al., 1994 [42]	To evaluate the effect of abstinence on bone mass and bone mineral metabolism in chronic alcoholics.	2 year longitudinal follow-up study	30 chronic alcoholic males	DPA	After 2 years of abstinence, Lumbar and femoral neck BMD increased in alcoholics and Baseline low osteocalcin increased after 1 year and 2 years of abstinence.
Peris et al., 1995 [41]	Association between vertebral facture and osteopenia in chronic alcoholics patients.	Cross-sectional study	76 chronic alcoholics and 62 age matched healthy males.	DPA	Chronic alcoholics frequently have traumas (68%) and vertebral fractures (36%) in spite of having a lumbar BMD above the fracture threshold.
Malik et al., 2009 [40]	To evaluate BMD according to alcohol consumption and sex.	Cross-sectional study	57 noncirrhotic alcoholic patients	DEXA	24.3% of men and 5% of women had low BMD and 75.7% of the men and 90% of the women had vitamin D insufficiency or deficiency.
**Studies on Patients with Genetic Hemochromatosis (GH) or Wilson Disease (WD)**					
**Author, Year**	**Study Aim**	**Study Design**	**Study Population**	**Method to Diagnosis Osteoporosis**	**Outcome**
Sinigaglia et al., 1997 [43]	To evaluate the prevalence and risk factor of osteoporosis in GH	Cross sectional study	32 patients with histologically proven GH	DEXA	Osteoporosis is observed in 28% and osteoporosis is highly associated with degree of iron overload (OR 3.23 (1.09–9.58))
Guggenbuhl et al., 2005 [44]	To assess BMD and bone remodeling in patients with GH	Retrospective study	38 men with HFE-related GH	DEXA	Osteopenia was observed in 78.9% of patients and osteoporosis in 34.2% that cannot solely be explained by hypogonadism or cirrhosis
Valenti et al., 2009 [45]	To identify the prevalence, clinical characteristics and genetic background associated with osteoporosis in patients with HHC	Retrospective study	87 patients with HHC	DEXA	Osteoporosis was identified in 25.3%, and osteopenia in 41.4% patients regardless of genetic background. Lumbar spine osteoporosis was independently associated with lower BMI (OR 0.73 (0.54–0.94)), total ALP (OR 1.17 (1–1.39)), and the amount of iron removed (OR 1.53 (1–2.5)).
Quemeneur et al., 2014 [47]	To assess the prevalent fractures, BMD and related risk factors in patients with WD.	Prospective cross-sectional study	85 patients with WD	DEXA	Prevalent peripheral fractures were presented in 51%, and vertebral fracture in 8% patients. Patients with severe neurological involvement, low BMI, old age are at risk factors for vertebral fractures
Weiss et al., 2015 [46]	Comparison of BMD between adult WD and healthy control population	Cross sectional study	148 adult WD patients and 345 age and gender matched control subjects	DEXA	Osteoporosis (8.8% vs. 4.1%) and osteopenia (50.0% vs. 41.2%) is significantly more prevalent in patient with WD than healthy population. There was no significant correlation between BMD and any of the WD disease parameters (e.g., the severity of liver disease), lab results, type of treatment or known osteoporosis risk factors.

PBC, Primary biliary cholangitis; PSC, primary sclerosing cholangitis; DEXA, dual energy X-ray absorptiometry; BMI, body mass index; OR, Odds ratio; HR, hazard ratio; BMD, body mineral density; RR, relative risk; CHC, Chronic hepatitis C; CHB, chronic hepatitis B; ETV, entecavir; TDF, Tenofovir; ALP, alkaline phosphatase; PTH, parathyroid hormone; LC, Liver cirrhosis; IGF-1, insulin-like growth factor-1; DPA, dual-photon absorptiometry; CTP, Child-turcotte-Pugh; OLT, orthotopic liver transplantation; NAFLD, non-alcoholic fatty liver disease; ALT, alanine aminotransferase; CRP, c-reactive protein; NASH, non-alcoholic steatohepatits; GH, genetic hemochromatosis; WD, Wilson disease; HHC, Hereditary hemochromatosis.

**Table 2:** Representative clinical studies for sarcopenia in chronic liver disease.

Studies on Patients with Non-Alcoholic Fatty Liver Disease (NAFLD)					
Author, Year	Study Aim	Study Design	Study Population	Method to Diagnosis Sarcopenia	Outcome
Hong et al., 2014 [100]	Relationship between sarcopenia and NAFLD	Cross-sectional study	452 subjects	DEXA	Lower muscle mass increased the risk of NAFLD (OR 5.16 (1.63–16.33)).
Lee et al., 2015 [99]	Association between sarcopenia and NAFLD or NASH	Cross-sectional study	15,132 subjects	DEXA	There was independent association between sarcopenia and NAFLD after adjusting for confounding factors related to obesity or insulin resistance (ORs 1.18 to 1.22)
Carias et al., 2016 [101]	Association between sarcopenic obestiy and NASH in patients with LC	Retrospective study	207 patients with LC	L3–L4 skeletal muscle mass on CT imaging, DEXA	NASH is independent predictor of sarcopenia obesity in patients with LC (OR 6.03 (1.44–25.26))
Koo et al., 2017 [102]	Association between sarcopenia and histological severity of NAFLD	Prospective cross-sectional study	309 patients (including 240 biopsy proven NAFLD patients)	BIA	Sarcopenia was significantly associated with NASH (OR 2.28 (1.12–4.30)) and significant fibrosis (OR 2.05 (1.01–4.16)).
Kim et al., 2018 [104]	Effect of skeletal muscle mass changes on NAFLD	7-year longitudinal cohort study	10,534 subjects without baseline NAFLD and 2631 subjects with baseline NAFLD	BIA	Increases in relative skeletal muscle mass over time may lead to benefits either in the development of NAFLD (aHR 0.44 (0.38–0.51)) or the resolution of existing NAFLD (aHR 2.09 (1.02–4.28)).
Gan et al., 2020 [103]	Associations of NAFLD with low muscle mass, low muscle strength, sarcopenia, and sarcopenic obesity	Cross-sectional study	5132 participants (including 1088 patients with NAFLD)	DEXA	Low muscle mass (OR 2.57 (2.03–3.25)), low muscle strength (OR 1.47 (1.21–1.80)), sarcopenia (OR 3.91 (2.90–5.28)), sarcopenic obesity (OR 1.42 (7.14–15.22)) were positively and associated with NAFLD.
**Studies on Patients with Liver Cirrhosis (LC)**					
**Author, Year**	**Study Aim**	**Study Design**	**Study Population**	**Method to Diagnosis Sarcopenia**	**Outcome**
Tandon et al., 2012 [105]	Prevalence of sarcopenia and clinical significance of sarcopenia in patients with cirrhosis listed for LT	Retrospective study	142 LC patients waiting for LT	L3 skeletal muscle on CT and MRI images. DEXA	The prevalence of sarcopenia was 41%, and sarcopenia is independent predictor of mortality (HR 2.36 (1.23–4.53))
Montano-Loza et al., 2012 [106]	Incidence of sarcopenia, association between sarcopenia and mortality and prognosis in LC patients	Prospective study	112 patients with LC	L3 skeletal muscle on CT images	The incidence of sarcopenia is 40%. Sarcopenia is associated with mortality in patients with cirrhosis. (HR 2.21)
Merli et al., 2013 [107]	The relationship between muscle depletion and hepatic encephalopathy (HE)	Prospective study	300 patients with LC	Mid-Arm-Muscle-Circumference, Triceps Skinfold-Thickness, Handgrip strength	HE were significantly higher in cirrhotic patients with muscle depletion or decreased muscle strength. (30% vs. 15%, and 29% vs. 16%, respectively)
Kim et al., 2014 [108]	The association between sarcopenia and long term mortality in LC patients with ascites	Retrospective study	65 patients with LC	psoas muscle thickness on CT images	Sarcopenia is an independent useful predictor for long-term mortality in cirrhotic patients with ascites. (HR 0.812 (0.684–0.965)).
Durand et al., 2014 [109]	Prognostic value of muscle atrophy in cirrhosis	Retrospective study	562 patients with LC	TPMT on CT image	TPMT/height on CT predicted mortality in cirrhotic patients, independent of the MELD and MELD-Na scores. (HR 0.86 (0.78–0.94) and HR 0.87 (0.79–0.95)).
Hanai et al., 2015 [110]	The prevalence of sarcopenia in patients with LC, Association between sarcopenia and outcomes.	Retrospective study	130 patients with LC	L3 skeletal muscle on CT images	The prevalence of sarcopenia was 68% and Sarcopenia is significantly associated with mortality in patients with LC. (HR 3.03)
Montano-Loza et al., 2015 [111]	The impact of sarcopenia in cirrhosis and mortality prediction of inclusion muscularity assessment within model for end-stage liver disease (MELD)	Retrospective study	669 patients with LC	L3 skeletal muscle on CT images	Sarcopenia (HR 0.97 (0.96–0.99)) were associated with mortality. Modification of MELD to include sarcopenia is associated with improved prediction of mortality in patients with cirrhosis, primarily in patients with low MELD scores. (C-statistics 0.73 (0.70–0.77)).
Hanai et al., 2016 [112]	The relationship between time-course change in skeletal muscle and the prognosis of patients with LC	Retrospective study	149 patients with LC	L3 skeletal muscle on CT images	The relative change in skeletal muscle area per year (>−3.1%) is useful for predicting mortality in patients with liver cirrhosis. (HR 2.73 (1.43–5.44)).
Nardelli et al., 2017 [113]	The association between sarcopenia and HE after TIPS	Prospective study	46 patients with LC	L3 skeletal muscle on CT images	Sarcopenia is independently associated with the development of HE after TIPS (subdistribution HR, 31.3 (4.5–218.07)).
Kang et al., 2018 [114]	Impact of sarcopenia to the conventional prognostic factors (MELD, CTP, HVPG)	Retrospective study	452 patients with LC	L3 skeletal muscle on CT images	The prevalence of sarcopenia was 42%. Sarcopenia is associated with mortality in compensated and early decompensated cirrhosis. Existing conventional prognostic factors had limited value in severe sarcopenia. (MELD, *p* = 0.182; CTP, *p* = 0.187; HVPG, *p* = 0.077).
**Studies on Patients Who Underwent Liver Transplantation**					
**Author, Year**	**Study Aim**	**Study Design**	**Study Population**	**Method to Diagnosis Sarcopenia**	**Outcome**
Englesbe et al., 2010 [115]	Association between sarcopenia and mortality after LT	Retrospective study	163 patients undergoing LT	psoas muscle at L4 vertebra on CT images	Total psoas area strongly correlates with mortality after LT (HR 0.274 (0.141–0.531)).
Kaido et al., 2013 [116]	Impact of sarcopenia on survival after LT	Retrospective study	124 undergoing LT	BIA	Low skeletal muscle mass was an independent risk factor for death after transplantation. (OR 4.846 (2.092–11.790))
Krell et al., 2013 [117]	Association between sarcopenia and serious infection after LT	Retrospective study	207 patients undergoing LT	TPA on CT images	Recipient age (HR 1.04), pre-transplant total psoas muscle area (HR 0.38) and pre-transplant total bilirubin level (HR 1.05) were independently associated with the risk of developing severe infections.
Tsien et al., 2014 [118]	The effect of changes in skeletal muscle mass on outcomes after LT	Prospective study	53 patients undergoing LT	Psoas and paraspinal muscles on CT images	Loss of muscle mass post-OLT increased risk of diabetes mellitus (HR 3.1 (1.01–9.38)) and a trend toward higher mortality.
Montano-Loza et al., 2014 [119]	Impact of muscle depletion on morbidity or mortality after LT	Retrospective study	248 patients undergoing LT	L3 skeletal muscle on CT images	Sarcopenia is predictive of longer hospital stays (40 ± 4 vs. 25 ± 3 days) and a higher risk of perioperative bacterial infection (26% vs. 15%) after LT.
Masuda et al., 2014 [120]	Impact of sarcopenia on mortality and sepsis after living donor LT	Retrospective study	204 patients undergoing LT	Psoas muscle at L3 vertebra on CT images	Sarcopenia is an independent predictor of mortality (HR 2.06) and sepsis after LDLT (HR 5.31)
Hamaguchi et al., 2014 [121]	Impact of quality and quantity of skeletal muscle on preoperative CT on outcomes after LT	Retrospective study	200 patients undergoing LT	IMAC and PMI on CT images	High IMAC (OR 3.898 (2.025–7.757)) and low PMI (OR 3.635 (1.896–7.174) were independent risk factors for death after LT.
Kalafateli et al., 2016 [122]	The impact of sarcopenia on post LT outcomes	Retrospective study	232 patients undergoing LT	L3-PMI on CT images	Sarcopenia were independent predictors of Hospital stay >20 days (OR 0.996 (0.994–0.999)) and 12 month mortality (OR 0.996 (0.992–0.999))
**Studies on Patients with Hepatocellular Carcinoma (HCC)**					
**Author, Year**	**Study Aim**	**Study Design**	**Study Population**	**Method to Diagnosis Sarcopenia**	**Outcome**
Meza-Junco et al., 2013 [123]	Frequency and prognostic significance of sarcopenia in patients with HCC.	Prospective study	116 patients with HCC	L3 SMI on CT images	Sarcopenia is present in 30% of patients with HCC and independent risk factor for mortality. (HR, 2.04) with median survival of 16 ± 6 (vs. 28 ± 3 months in nonsarcopenic).
Harimoto et al., 2013 [124]	The effect of sarcopenia on outcomes after partial hepatectomy for HCC	Retrospective study	186 patients with HCC	L3 skeletal muscle on CT images	5-year overall survival rate and 5-year recurrence-free survival rate was 71% vs. 83.7% and 13% vs. 33.2% in patients with and without sarcopenia, respectively. Sarcopenia was predictive of an overall survival (HR 3.27 (1.39–7.69)) and recurrence free survival (HR 0.97 (0.95–1.00)).
Fujiwara et al., 2015 [125]	Impact of body composition on HCC	Retrospective study	1257 patients with different stages of HCC	SMI, mean MA, visceral adipose tissue index, subcutaneous adipose tissue index, VSR via on CT images	Sarcopenia (HR 1.52 (1.18–1.96)), intramuscular fat deposition (HR 1.34 (1.05–1.71)), and visceral adiposity (HR 1.35 (1.09–1.66)) independently predict mortality in patients with HCC.

NAFLD, non-alcoholic fatty liver disease; DEXA, dual energy X-ray absorptiometry; OR, odds ratio; NASH, non-alcoholic steatohepatitis; LC, liver cirrhosis; BIA, bioelectrical impedance analysis; HR, hazard ratio; LT, liver transplantation; HE, hepatic encephalopathy; TIPS, trans-jugular intrahepatic portosystemic shunt; MELD, model for end-stage liver disease; HVPG, hepatic venous pressure gradient; PMI, psoas muscle index; TPMT, transversal psoas muscle thickness; IMAC, intramuscular adipose tissue content; PMI, psoas muscle mass index; TPA, total psoas area; SMI, skeletal muscle index; HCC, Hepatocelluar carcinoma; MA, muscle attenuation; VSR, visceral to subcutaneous adipose tissue area ratios.

**Table 3:** Representative clinical studies for osetosarcopenia in chronic liver disease.

Author, Year [Reference]	Study Aim	Study Design	Study Population	Method to Diagnosis Osteosarcopenia	Outcome
Santos et al., 2016 [188]	To evaluate whether handgrip strength, bone, and liver tests may be useful as predictors of bone disease in outpatients with LC	Prospective study	129 patients with LC	DEXA, dynamometer	For lumbar spine, only low handgrip strength and high PTH levels were clearly related to low T scores.
Hayashi et al., 2018 [185]	Association between sarcopenia and osteoporosis in patients with CLD	Retrospective study	112 CLD patients including 40 cirrhotic patients	BIA and DEXA	The sarcopenia rate was 13%, and the osteoporosis and osteopenia rates were 17% and 65%, respectively. Sarcopenia was significantly associated with the BMD of the lumbar spine and the femur neck. Sarcopenia (OR 6.16) and cirrhosis (OR 15.8) were independent risk factors for osteoporosis.
Hayashi et al., 2018 [186]	Association between loss of skeletal muscle mass and clinical factors such as osteoporosis in patients with chronic liver disease.	Cross-sectional study	112 HCC patients undergoing hepatectomy	DEXA	The T-score and PeakVO2 was significantly lower in the low skeletal mass index (SMI) group. T-score (OR 3.508 (1.074–11.456)) and PeakVO2 (OR 3.512 (1.114–11.066)) were significantly related to SMI, independent of age and sex.
Bering et al., 2018 [187]	To assess the prevalence of low BMD and its association with body composition, muscle strength, and nutritional status in patients with CHC.	Prospective cross-sectional study	104 patients with CHC	DEXA	Low BMD, low muscle strength, pre-sarcopenia, sarcopenia, and sarcopenic obesity were presented in 34.6%, 27.9%, 14.4%, 8.7%, and 3.8% of the patients, respectively. Appendicular skeletal muscle mass is an independent predictor of BMD in CHC. Sarcopenia was independently associated with bone mineral content and malnutrition.
Saeki et al., 2020 [189]	Association between osteosarcopenia and frailty in patients with CLD	Cross-sectional study	291 patients with CLD	DEXA	49 (16.8%) and 81 (27.8%) had osteosarcopenia and frailty, respectively. Frailty was an independently associated with osteosarcopenia (OR 9.837), and vice versa (OR 10.069) and increased the risk of vertebral fracture in patients with CLD.

CLD, chronic liver disease; BIA, bioelectrical impedance analysis; DEXA, dual energy X-ray absorptiometry; BMD, bone mineral density; OR, odds ratio; HCC, hepatocellular carcinoma; SMI, skeletal muscle index; CHC, chronic hepatitis C; LC, liver cirrhosis; PTH, parathyroid hormone.

**Table 4:** Pharmacologic treatments of osteoporosis, sarcopenia, and osteosarcopenia.

Disorders	Effect	Agents
Osteoporosis	Inhibit bone resorption	Calcium
	Inhibit bone resorption	Vitamin D
	Inhibit bone resorption	Calcitonin
	Inhibit bone resorption	SERMs (ex, raloxifene)
	Inhibit bone resorption	Bisphosphates (ex, alendronate, zoledronic acid, ibandronate)
	Inhibit bone resorption	Anti-RANKL antibody (ex, denosumab)
	Activate bone formation	PTH (ex, teriparatide)
	Activate bone formation	Sclerostin inhibitors (ex, romosozumab)
Sarcopenia	Reduce Ammonia	L-ornithine L-aspartate
	Reduce Ammonia	Rifaximin
	Increase muscle protein synthesis	Testosterone
	Increase muscle protein synthesis	Myostatin antagonists (ex, follistatin)
	Increase muscle protein synthesis	IGF-1 antagonist
Osteosarcopenia	Inhibit bone resorption Increase muscle protein synthesis (insulin sensitivity)	Anti-RANKL antibody (ex, denosumab)
	Activate bone and muscle formation	SARMs (ex, VK5211)
	Activate bone and muscle formation	Myostatin antagonists (ex, ACE-031)

SERMs; selective estrogen receptor modulators; RANKL, receptor-activator of nuclear factor kappa ligand; PTH, parathyroid hormones; SARMs, Selective androgen receptor modulator.

## Data Availability

Data is contained within the article.
